# Supplementary material for: Immunogenicity and safety of the MF59-adjuvanted seasonal influenza vaccine in non-elderly adults: A systematic review and meta-analysis
Source: PLoS One. 2024 Dec 30;19(12):e0310677. doi: 10.1371/journal.pone.0310677 (PMC11684710; doi:10.1371/journal.pone.0310677)
Supplement: S15 Fig — (DOCX) [file pone.0310677.s015.docx]

**S15 Fig. Funnel plot of difference in seroconversion rates towards vaccine-like B strains 3–4 weeks after one dose of the MF59-adjuvanted or non-adjuvanted seasonal influenza vaccines in non-elderly adults.**


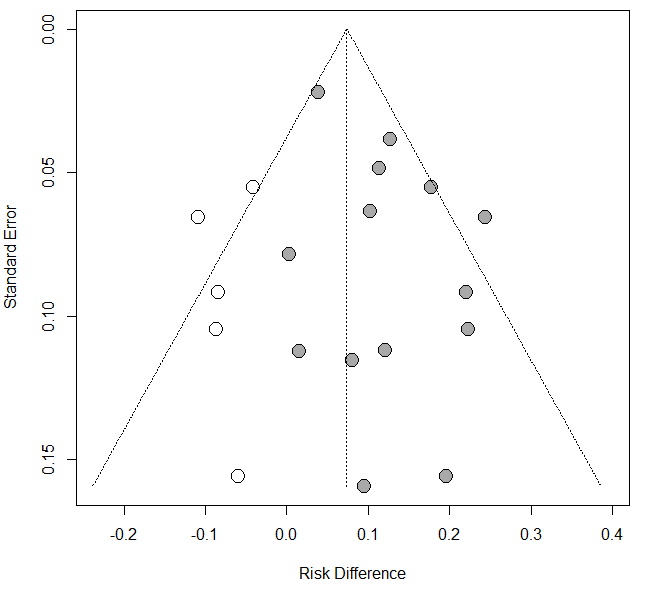


Observed studies Imputed studies Adjusted random-effects estimate: 7.4% (95% CI: 2.3%, 12.5%)
