# Supplementary material for: Immunogenicity and safety of the MF59-adjuvanted seasonal influenza vaccine in non-elderly adults: A systematic review and meta-analysis
Source: PLoS One. 2024 Dec 30;19(12):e0310677. doi: 10.1371/journal.pone.0310677 (PMC11684710; doi:10.1371/journal.pone.0310677)

**S45 Fig. Frequency of serious adverse events during after one dose of the MF59-adjuvanted seasonal influenza vaccine in non-elderly adults.**


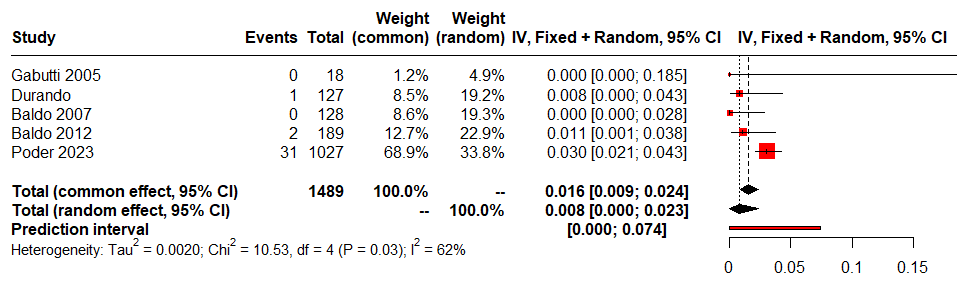

Supplement: S45 Fig — (DOCX) [file pone.0310677.s045.docx]
