# Supplementary material for: Immunogenicity and safety of the MF59-adjuvanted seasonal influenza vaccine in non-elderly adults: A systematic review and meta-analysis
Source: PLoS One. 2024 Dec 30;19(12):e0310677. doi: 10.1371/journal.pone.0310677 (PMC11684710; doi:10.1371/journal.pone.0310677)
Supplement: S2 Table — (DOCX) [file pone.0310677.s048.docx]

**S2 Table. Algorithm for the research performed on 16 February 2024, by citation database.**

| **Database** | **Script** | **Records retrieved** |
| --- | --- | --- |
| MEDLINE and Biological Abstracts via Ovid | 1. (fluad* or MF59* or MF 59*).mp. | 1431 |
|  | 1. exp Influenza Vaccines/ or influenza vaccin*.mp. or ((influenza or flu*) adj5 (vaccin* or immuni* or innoculat*)).mp. | 59139 |
|  | 1. influenza.mp. or exp Influenza, Human/ | 191433 |
|  | 1. exp Vaccines/ or vaccin*.mp. or exp Viral Vaccines/ or immuni*.mp. or Vaccines, Subunit/ or Vaccines, Synthetic/ | 1201645 |
|  | 1. 3 and 4 | 76270 |
|  | 1. exp Adjuvants, Immunologic/ or adjuvant*.mp. or squalene*.mp. or Polysorbate*.mp. or Emulsion*.mp. | 537308 |
|  | 1. (2 or 5) and 6 | 7281 |
|  | 1. 1 or 7 | 7919 |
|  | 1. exp Adult/ | 9410260 |
|  | 1. exp Middle Aged/ | 4744043 |
|  | 1. exp Young Adult/ | 1021927 |
|  | 1. ((working age* or middle age*) adj3 (people* or person* or adult* or women* or men*)).tw. | 705992 |
|  | 1. or/9-12 | 9421561 |
|  | 1. 8 and 13 | **1549** |
| Web of Science | 1. TS=(fluad* or MF59*) | 1001 |
|  | 1. TS=((influenza vaccin*) OR ((infuenza* or flu*) near/5 (vaccin* or immuni* or innoculat*))) | 51467 |
|  | 1. TS=influenza* | 155112 |
|  | 1. TS=(vaccin* or immuni*) | 793783 |
|  | 1. #4 AND #3 | 61421 |
|  | 1. #5 OR #2 | 64362 |
|  | 1. TS=(adjuvant* or squalene* or polysorbate* or emuls*) | 372181 |
|  | 1. #7 AND #6 | 5406 |
|  | 1. #8 OR #1 | 5731 |
|  | 1. TS=(working age* adult or young adult* or middle age* adult* or working age* women* or young women* or middle age* women* or working age* men* or young men* or middle age* men* or working age* people* or young people* or middle age* people*) | 718653 |
|  | 1. #9 AND #10 | **261** |
| Cochrane Library | 1. fluad* or MF59* or MF 59* | 1872 |
|  | 1. MeSH descriptor: [Influenza Vaccines] explode all trees | 2104 |
|  | 1. MeSH descriptor: [Influenza, Human] explode all trees | 3545 |
|  | 1. MeSH descriptor: [Vaccines] in all MeSH products | 17306 |
|  | 1. #3 AND #4 | 2322 |
|  | 1. #2 OR #5 | 2963 |
|  | 1. MeSH descriptor: [Adjuvants, Immunologic] explode all trees | 2800 |
|  | 1. #6 AND #7 | 312 |
|  | 1. #1 OR 8 | 2069 |
|  | 1. MeSH descriptor: [Adult] explode all trees | 611867 |
|  | 1. MeSH descriptor: [Middle Aged] explode all trees | 404324 |
|  | 1. MeSH descriptor: [Young Adult] explode all trees | 95088 |
|  | 1. #10 OR #11 OR #12 | 611867 |
|  | 1. #9 AND #13 | **896** |
| ClinicalTrials.gov | 1. MF59 OR ADJUVANTED \| Completed Studies \| Influenza \| Adult | **174** |
