# Supplementary material for: Immunogenicity and safety of the MF59-adjuvanted seasonal influenza vaccine in non-elderly adults: A systematic review and meta-analysis
Source: PLoS One. 2024 Dec 30;19(12):e0310677. doi: 10.1371/journal.pone.0310677 (PMC11684710; doi:10.1371/journal.pone.0310677)
Supplement: S3 Table — (DOCX) [file pone.0310677.s049.docx]

**S3 Table. List of excluded studies with reasons.**

| **Study** | **Reason for exclusion** |
| --- | --- |
| Menegon 1999 | Ineligible population (predominantly older adults) |
| Amendola 2001 | Ineligible vaccine (virosomal) |
| Baldo 2006 | Ineligible population (older adults) |
| Del Giudice 2006 | Ineligible outcomes |
| Iorio 2006 | Ineligible population (predominantly older adults) |
| Werba 2008 | Ineligible population and outcomes |
| Herbinger 2014 | Experimental formulation of the MF59-adjuvanted influenza vaccine |
| Kumar 2014 | Redundant record (conference abstract of the included full article) |
| Fernández-Ruiz 2015 | Ineligible outcomes; no specific data on the MF59-adjuvanted seasonal influenza vaccine |
| Pérez-Romero 2015 | Ineligible vaccine (pandemic formulation) |
| Caso 2016 | Ineligible outcomes |
| Sánchez de Prada 2020 | Ineligible vaccine and population (MF59-adjuvanted seasonal influenza vaccine used only in older adults) |

**References:**

Amendola A, Boschini A, Colzani D, Anselmi G, Oltolina A, Zucconi R, et al. Influenza vaccination of HIV-1-positive and HIV-1-negative former intravenous drug users. J Med Virol. 2001;65(4):644-8.

Baldo V, Baldovin T, Floreani A, Minuzzo M, Trivello R. Response to influenza vaccine in people with non-protective HI antibody titers. Eur J Epidemiol. 2006;21(11):843-5.

Caso F, Ramonda R, Del Puente A, Darda MA, Cantarini L, Peluso R, et al. Influenza vaccine with adjuvant on disease activity in psoriatic arthritis patients under anti-TNF-α therapy. Clin Exp Rheumatol. 2016;34(3):507-12.

Del Giudice G, Fragapane E, Bugarini R, Hora M, Henriksson T, Palla E, et al. Vaccines with the MF59 adjuvant do not stimulate antibody responses against squalene. Clin Vaccine Immunol. 2006;13(9):1010-3.

Fernández-Ruiz M, Lumbreras C, Arrazola MP, López-Medrano F, Andrés A, Morales JM, et al. Impact of squalene-based adjuvanted influenza vaccination on graft outcome in kidney transplant recipients. Transpl Infect Dis. 2015;17(2):314-21.

Herbinger KH, von Sonnenburg F, Nothdurft HD, Perona P, Borkowski A, Fragapane E, et al. A phase II study of an investigational tetravalent influenza vaccine formulation combining MF59®: adjuvanted, pre-pandemic, A/H5N1 vaccine and trivalent seasonal influenza vaccine in healthy adults. Hum Vaccin Immunother. 2014;10(1):92-9.

Iorio AM, Camilloni B, Basileo M, Guercini F, Conti S, Ferrante F, et al. Influenza vaccination in patients on long-term anticoagulant therapy. Vaccine. 2006;24(44-46):6624-8.

Kumar, D, Campbell P, Hidalgo L, Hoschler K, Al-Dabbagh M, Humar A. Randomized trial of a MF-59 adjuvanted influenza vaccine in kidney transplant recipients. Abstract# D2380. Transplantation 2014;98:767-8.

Menegon T, Baldo V, Bonello C, Dalla Costa D, Di Tommaso A, Trivello R. Influenza vaccines: antibody responses to split virus and MF59-adjuvanted subunit virus in an adult population. Eur J Epidemiol. 1999;15(6):573-6.

Pérez-Romero P, Bulnes-Ramos A, Torre-Cisneros J, Gavaldá J, Aydillo TA, Moreno A, et al. Influenza vaccination during the first 6 months after solid organ transplantation is efficacious and safe. Clin Microbiol Infect. 2015;21(11):1040.e11-8.

Sánchez de Prada L, Sanz Muñoz I, Castrodeza Sanz J, Ortiz de Lejarazu Leonardo R, Eiros Bouza JM. Adjuvanted influenza vaccines elicits higher antibody responses against the A(H3N2) subtype than non-adjuvanted vaccines. Vaccines (Basel). 2020;8(4):704.

Werba JP, Veglia F, Amato M, Baldassarre D, Massironi P, Meroni PL, et al. Patients with a history of stable or unstable coronary heart disease have different acute phase responses to an inflammatory stimulus. Atherosclerosis. 2008;196(2):835-40.
