# Supplementary material for: Immunogenicity and safety of the MF59-adjuvanted seasonal influenza vaccine in non-elderly adults: A systematic review and meta-analysis
Source: PLoS One. 2024 Dec 30;19(12):e0310677. doi: 10.1371/journal.pone.0310677 (PMC11684710; doi:10.1371/journal.pone.0310677)
Supplement: S4 Table — (DOCX) [file pone.0310677.s050.docx]

**S4 Table. Risk of bias (RoB) assessment of the selected randomized trials.**

| **Study [Ref]** | **D1** | **D2** | **D3** | **D4** | **D5** | **Overall** |
| --- | --- | --- | --- | --- | --- | --- |
| Frey 2003 [42] | SC | Low | Low | Low | Low | SC |
| Magnani 2005 [43] | High | Low | Low | High | Low | High |
| Pollok 2004 [45] | SC | Low | High | Low | SC | High |
| Gabutti 2005 [46] | High | Low | Low | Low | Low | High |
| Durando 2008 [48] | SC | Low | Low | Low | Low | SC |
| Baldo 2007 [50] | Low | Low | Low | Low | Low | Low |
| Baldo 2012 [65] | Low | Low | Low | Low | Low | Low |
| Kumar 2016 [54] | Low | Low | Low | Low | Low | Low |
| Spensieri 2016 [56] | Low | Low | Low | Low | Low | Low |
| Noh 2016 [59] | Low | Low | Low | Low | Low | Low |
| Natori 2017 [60] | SC | Low | SC | Low | Low | SC |
| Mombelli 2024 [62] | Low | Low | Low | Low | Low | Low |
| Poder 2003 [63] | Low | Low | Low | Low | Low | Low |

SC, some concerns.

D1: Bias due to randomization.

D2: Bias due to deviations from intended intervention.

D3: Bias due to missing data.

D4: Bias due to outcome measurement.

D5: Bias due to selection of reported result.
