# Supplementary material for: Immunogenicity and safety of the MF59-adjuvanted seasonal influenza vaccine in non-elderly adults: A systematic review and meta-analysis
Source: PLoS One. 2024 Dec 30;19(12):e0310677. doi: 10.1371/journal.pone.0310677 (PMC11684710; doi:10.1371/journal.pone.0310677)
Supplement: S5 Table — (DOCX) [file pone.0310677.s051.docx]

**S5 Table. Risk of bias (RoB) assessment of the selected non-randomized trials.**

| **Study [Ref]** | **D1** | **D2** | **D3** | **D4** | **D5** | **D6** | **D7** | **Overall** |
| --- | --- | --- | --- | --- | --- | --- | --- | --- |
| Iorio 2003 [44] | Moderate | Moderate | Low | Low | Low | Low | Low | Moderate |
| Camilloni 2009 [47] | High | High | Low | Low | Low | Low | Low | High |
| Iorio 2012 [52] | High | High | Low | Low | Low | Low | Low | High |
| Fabbiani 2013 [53] | High | High | Low | Low | Low | High | Low | High |
| Kazmin 2023 [61] | Low | Low | Low | Low | Low | Low | Low | Low |

D1: Bias due to confounding.

D2: Bias due to selection of participants.

D3: Bias in classification of interventions.

D4: Bias due to deviations from intended interventions.

D5: Bias due to missing data.

D6: Bias in measurement of outcomes.

D7: Bias due to selection of reported result.
