# Supplementary material for: Immunogenicity and safety of the MF59-adjuvanted seasonal influenza vaccine in non-elderly adults: A systematic review and meta-analysis
Source: PLoS One. 2024 Dec 30;19(12):e0310677. doi: 10.1371/journal.pone.0310677 (PMC11684710; doi:10.1371/journal.pone.0310677)
Supplement: S6 Table — (DOCX) [file pone.0310677.s052.docx]

**S6 Table. Extracted data on the comparison of seroconversion and seroprotection rates and geometric mean titer ratios against vaccine-like strains 3–4 weeks after one dose of the MF59-adjuvanted or non-adjuvanted seasonal influenza vaccines in non-elderly adults, by strain, presence of immunosuppressive conditions and serological parameter.**

| **Virus** | **Immuno-suppression** | **Seroconversion rate, % (n/N)** | | | | **Seroprotection rate, % (n/N)** | | | **GMTR aTIV/aQIV vs TIV/QIV (95% CI or p)** | **Ref** |
| --- | --- | --- | --- | --- | --- | --- | --- | --- | --- | --- |
|  |  | **aTIV/aQIV** | **TIV/QIV** | **P** | **aTIV/aQIV** | | **TIV/QIV** | **P** |  |  |
| A(H1N1) | No | 61 (88/145) | 53 (80/150) | ns | 99 (144/145)^a^ | | 95 (143/150)^a^ | ns | 1.12 (ns) | [42] |
|  |  | 53.8 (14/26) | NA | NA | 96.2 (25/26)^b^ | | NA | NA | NA | [64] |
|  |  | 57.5 (69/120) | 55.9 (66/118) | ns | 97.5 (117/120)^b^ | | 96.6 (114/118)^b^ | ns | 1.06 (0.59; 1.53)^c^ | [50] |
|  |  | 63 (103/163) | 47 (73/156) | <0.01 | 87 (142/163)^b^ | | 74 (115/156)^b^ | <0.01 | 1.96 (<0.001) | [65] |
|  |  | 62.5 (10/16) | NA | NA | 75.0 (12/16)^b^ | | NA | NA | NA | [52] |
|  |  | 64.7 (11/17) | 83.3 (15/18) | ns | 100 (17/17)^b^ | | 88.9 (16/18)^b^ | ns | 2.46 (ns) | [56] |
|  |  | 61 (49/80) | 59 (42/71) | ns | 94 (75/80)^b^ | | 90 (64/71)^b^ | ns | 2.01 (0.005) | [48] |
|  |  | 67.9 (19/28) | NA | NA | 100 (28/28) | | NA | NA | NA | [61] |
|  |  | 81.2 (798/983) | 76.8 (756/985) | <0.05 | 99.7 (1024/1027)^b^ | | 99.2 (1008/1016)^b^ | ns | 1.24 (1.14; 1.34) | [63] |
|  | Yes | 76 (34/45) | 55 (28/50) | 0.046 | 89 (40/45)^b^ | | 78 (39/50)^b^ | ns | 1.4 (0.7; 2.7) | [45] |
|  |  | 52 (23/44) | 33 (13/40) | ns | 70 (31/44)^b^ | | 53 (21/40)^b^ | ns | 1.76 (<0.05) | [44] |
|  |  | 61 (11/18) | 68 (13/19) | ns | 83 (15/18)^b^ | | 100 (19/19)^b^ | ns | 0.42 (ns) | [46] |
|  |  | 45 (15/33) | 41 (20/49) | ns | 94 (31/33)^b^ | | 90 (44/49)^b^ | ns | 2.36 (0.003) | [48] |
|  |  | 45.2 (14/31) | 48.3 (14/29) | 0.81 | 83.9 (26/31)^b^ | | 86.2 (25/29)^b^ | 0.80 | 1.63 (0.34) | [54] |
|  |  | 46.3 (31/67) | 13.8 (8/58) | <0.01 | 85.1 (57/67)^b^ | | 69.0 (40/58)^b^ | 0.05 | 2.23 (<0.01) | [59] |
|  |  | 31.4 (11/35) | 21.9 (7/32) | 0.38 | 57.1 (20/35)^b^ | | 59.4 (19/32)^b^ | 0.85 | 1.11 (0.65) | [60] |
|  |  | 27 (55/205) | 17 (34/198) | <0.05 | 85 (174/205)^b^ | | 80 (159/198)^b^ | ns | 1.42 (0.005) | [62] |
| A(H3N2) | No | 77 (112/145) | 75 (113/150) | ns | 94 (136/145)^a^ | | 91 (137/150)^a^ | ns | 1.22 (ns)^c^ | [42] |
|  |  | 52.5 (63/120) | 33.1 (39/118) | 0.02 | 75.0 (90/120)^b^ | | 57.6 (68/118)^b^ | 0.002 | 2.11 (1.65; 2.54) | [50] |
|  |  | 77 (126/163) | 60 (94/156) | <0.01 | 93 (152/163)^b^ | | 78 (122/156)^b^ | <0.001 | 2.21 (<0.001) | [65] |
|  |  | 50.0 (8/16) | NA | NA | 87.5 (14/16)^b^ | | NA | NA | NA | [52] |
|  |  | 82.4 (14/17) | 72.2 (13/18) | ns | 100 (17/17)^b^ | | 83.3 (15/18)^b^ | ns | 2.62 (ns) | [56] |
|  |  | 66 (53/80) | 69 (49/71) | ns | 96 (77/80)^b^ | | 97 (69/71)^b^ | ns | 1.24 (ns) | [48] |
|  |  | 89.3 (25/28) | NA | NA | 100 (28/28)^b^ | | NA | NA | NA | [61] |
|  |  | 63.6 (625/983) | 61.8 (609/985) | ns | 97.5 (1001/1027)^b^ | | 97.3 (989/1016)^b^ | ns | 1.10 (1.002; 1.21) | [63] |
|  | Yes | 76 (34/45) | 23 (29/50) | ns | 84 (38/45)^b^ | | 56 (28/50)^b^ | 0.003 | 3.5 (1.4; 8.7) | [45] |
|  |  | 61 (27/44) | 23 (9/40) | <0.01 | 82 (36/44)^b^ | | 55 (22/40)^b^ | <0.05 | 3.03 (<0.01) | [44] |
|  |  | 44 (8/18) | 53 (10/19) | ns | 72 (13/18)^b^ | | 74 (14/19)^b^ | ns | 0.68 (ns) | [46] |
|  |  | 54 (18/33) | 45 (22/49) | ns | 94 (31/33)^b^ | | 83 (40/49)^b^ | ns | 1.79 (ns) | [48] |
|  |  | 48.4 (15/31) | 34.5 (10/29) | 0.28 | 100 (31/31)^b^ | | 93.1 (27/29)^b^ | 0.23 | 1.13 (0.96) | [54] |
|  |  | 40.3 (27/67) | 10.3 (6/58) | <0.01 | 97.0 (65/67)^b^ | | 89.7 (52/58)^b^ | 0.14 | 1.62 (0.01) | [59] |
|  |  | 57.1 (20/35) | 40.6 (13/32) | 0.18 | 71.4 (25/35)^b^ | | 71.9 (23/32)^b^ | 0.97 | 1.34 (0.27) | [60] |
|  |  | 52 (106/205) | 35 (69/198) | <0.001 | 71 (146/205)^b^ | | 62 (122/198)^b^ | <0.05 | 1.57 (0.002) | [62] |
| B/Victoria | No | NA | NA | NA | 96.1 (25/26)^b^ | | NA | NA | NA | [47] |
|  |  | 62 (101/163) | 44 (69/156) | <0.01 | 67 (109/163)^b^ | | 49 (76/156)^b^ | <0.001 | 1.73 (<0.01) | [65] |
|  |  | 56.3 (9/16) | NA | NA | 81.2 (13/16)^b^ | | NA | NA | NA | [52] |
|  |  | 44.5 (437/983) | 40.6 (400/985) | ns | 94.4 (969/1027)^b^ | | 93.3 (948/1016)^b^ | ns | 1.00 (0.93; 1.07) | [63] |
|  | Yes | 72 (13/18) | 53 (10/19) | ns | 94 (17/18)^b^ | | 89 (17/19)^b^ | ns | 0.63 (ns) | [46] |
|  |  | 25 (52/205) | 13 (25/198) | <0.01 | 53 (109/205)^b^ | | 40 (80/198)^b^ | <0.05 | 1.30 (0.033) | [62] |
| B/Yamagata | No | 83 (120/145) | 71 (107/150) | ≤0.01 | 99 (144/145)^a^ | | 97 (146/150)^a^ | ns | 1.16 (ns) | [42] |
|  |  | 46.7 (56/120) | 36.4 (43/118) | ns | 69.2 (83/120)^b^ | | 61.0 (72/118)^b^ | ns | 1.50 (1.07; 1.92) | [50] |
|  |  | 64 (51/80) | 63 (45/71) | ns | 96 (77/80)^b^ | | 93 (66/71)^b^ | ns | 1.79 (0.023) | [48] |
|  |  | 70.6 (12/17) | 61.1 (11/18) | ns | 88.2 (15/17)^b^ | | 61.1 (11/18)^b^ | ns | 2.59 (ns) | [56] |
|  |  | 53.6 (15/28) | NA | NA | 57.1 (16/28)^b^ | | NA | NA | NA | [61] |
|  |  | 43.4 (427/983) | 41.0 (404/985) | ns | 95.9 (985/1027)^b^ | | 94.6 (961/1016)^b^ | ns | 1.06 (0.99; 1.13) | [63] |
|  | Yes | 52 (23/44) | 30 (12/40) | ns | 70 (31/44)^b^ | | 55 (22/40)^b^ | ns | 1.96 (<0.05) | [44] |
|  |  | 80 (36/45) | 58 (29/50) | 0.021 | 89 (40/45)^b^ | | 90 (45/50)^b^ | ns | 1.2 (0.6; 2.7) | [45] |
|  |  | 48 (16/33) | 47 (23/49) | ns | 94 (31/33)^b^ | | 90 (44/49)^b^ | ns | 1.57 (0.003) | [48] |
|  |  | 32.3 (10/31) | 24.1 (7/29) | 0.49 | 61.3 (19/31)^b^ | | 65.5 (19/29)^b^ | 0.73 | 1.48 (0.33) | [54] |
|  |  | 31.3 (21/67) | 6.9 (4/58) | <0.01 | 92.5 (62/67)^b^ | | 84.5 (49/58)^b^ | 0.15 | 1.26 (0.18) | [59] |
|  |  | 37.1 (13/35) | 25.0 (8/32) | 0.29 | 57.1 (20/35)^b^ | | 68.8 (22/32)^b^ | 0.32 | 1.24 (0.97) | [60] |

^a^ Hemagglutination inhibition assay titer ≥1:160; ^b^ Hemagglutination inhibition assay titer ≥1:40.

aQIV, quadrivalent MF59-adjuvanted seasonal influenza vaccine; aTIV, trivalent MF59-adjuvanted seasonal influenza vaccine; QIV, quadrivalent non-adjuvanted seasonal influenza vaccine; TIV, trivalent non-adjuvanted seasonal influenza vaccine; GMTR, geometric mean titer ratio; ns, non-significant at p<0.05; NA, not available.
