# Supplementary material for: Immunogenicity and safety of the MF59-adjuvanted seasonal influenza vaccine in non-elderly adults: A systematic review and meta-analysis
Source: PLoS One. 2024 Dec 30;19(12):e0310677. doi: 10.1371/journal.pone.0310677 (PMC11684710; doi:10.1371/journal.pone.0310677)
Supplement: S7 Table — (DOCX) [file pone.0310677.s053.docx]

**S7 Table. Absolute seroprotection rates towards vaccine-like strains 3–4 weeks after one dose of the MF59-adjuvanted seasonal influenza vaccine in non-elderly adults: A sensitivity analysis by including any hemagglutination inhibition titer threshold (both ≥ 1:40 and ≥ 1:160).**

| **Vaccine-like strain** | **k** | **I^2^, %** | **FE model, % (95% CI)** | **RE model, % (95% CI)** |
| --- | --- | --- | --- | --- |
| A(H1N1) | 16 | 93.8 | 97.5 (96.6, 98.2) | 90.7 (84.9, 95.8) |
| A(H3N2) | 16 | 92.3 | 94.2 (93.1, 95.3) | 90.9 (85.1, 95.5) |
| B | 17 | 95.5 | 88.2 (86.7, 89.6) | 82.7 (73.8, 90.1) |
