# Supplementary material for: Immunogenicity and safety of the MF59-adjuvanted seasonal influenza vaccine in non-elderly adults: A systematic review and meta-analysis
Source: PLoS One. 2024 Dec 30;19(12):e0310677. doi: 10.1371/journal.pone.0310677 (PMC11684710; doi:10.1371/journal.pone.0310677)
Supplement: S8 Table — (DOCX) [file pone.0310677.s054.docx]

**S8 Table. Meta-analysis of absolute and relative seroconversion and seroprotection rates** **towards vaccine-like strains 3–4 weeks after one dose of the MF59-adjuvanted** **or non-adjuvanted seasonal influenza vaccines: A sensitivity analysis by excluding studies with some elderly participants.**

| **Parameter** | **Vaccine-like strain** | **k** | **I^2^, %** | **FE model, % (95% CI)** | **RE model, % (95% CI)** |
| --- | --- | --- | --- | --- | --- |
| SCR | A(H1N1) | 14 | 89.1 | 72.5 (70.4, 74.7) | 62.0 (55.4, 68.3) |
|  | A(H3N2) | 13 | 82.2 | 65.4 (63.1, 67.6) | 65.2 (56.9, 73.1) |
|  | B | 13 | 91.0 | 52.1 (49.7, 54.4) | 58.7 (49.6, 67.6) |
| SPR | A(H1N1) | 12 | 92.5 | 98.7 (97.9, 99.3) | 92.4 (86.1, 97.1) |
|  | A(H3N2) | 12 | 87.9 | 96.2 (95.1, 97.2) | 92.3 (86.5, 96.7) |
|  | B | 13 | 92.2 | 90.8 (89.2, 92.2) | 85.6 (77.8, 92.0) |
| ΔSCR | A(H1N1) | 11 | 59.0 | 7.0 (4.0, 9.9) | 9.2 (2.4, 16.0) |
|  | A(H3N2) | 11 | 74.4 | 6.8 (3.6, 9.9) | 12.6 (4.6, 20.6) |
|  | B | 11 | 48.4 | 8.0 (4.7, 11.2) | 12.0 (6.4, 17.6) |
| ΔSPR | A(H1N1) | 11 | 61.8 | 3.4 (2.0, 4.8) | 4.5 (0.9, 8.0) |
|  | A(H3N2) | 11 | 78.5 | 4.9 (3.2, 6.6) | 9.6 (3.7, 15.4) |
|  | B | 11 | 41.7 | 4.2 (2.2, 6.2) | 4.8 (1.3, 8.2) |

FE, fixed effects; RE, random effects; SCR, seroconversion rate; SPR, seroprotection rate; ΔSCR: difference in seroconversion rates between subjects immunized with adjuvanted vs non-adjuvanted influenza vaccines; ΔSPR: difference in seroprotection rates between subjects immunized with adjuvanted vs non-adjuvanted influenza vaccines.
