# Supplementary material for: Immunogenicity and safety of the MF59-adjuvanted seasonal influenza vaccine in non-elderly adults: A systematic review and meta-analysis
Source: PLoS One. 2024 Dec 30;19(12):e0310677. doi: 10.1371/journal.pone.0310677 (PMC11684710; doi:10.1371/journal.pone.0310677)
Supplement: S9 Table — (DOCX) [file pone.0310677.s055.docx]

**S9 Table. Univariable meta-regression analysis to investigate sources of heterogeneity in relative seroconversion and seroprotection rates towards vaccine-like strains 3–4 weeks after one dose of the MF59-adjuvanted or non-adjuvanted seasonal influenza vaccines.**

| **Virus-like strain** | **Outcome** | **Predictor** | **Coefficient** | **Standard error** | **p** |
| --- | --- | --- | --- | --- | --- |
| A(H1N1) | ΔSCR | Sample size <100 | -0.072 | 0.071 | 0.31 |
|  |  | Low risk of bias | 0.002 | 0.058 | 0.97 |
|  |  | Industry sponsorship | -0.017 | 0.057 | 0.77 |
|  |  | Overlapping population | -0.019 | 0.070 | 0.79 |
|  |  | Immunosuppressed population | 0.079 | 0.048 | 0.10 |
|  | ΔSPR | Sample size <100 | -0.026 | 0.049 | 0.60 |
|  |  | Low risk of bias | 0.005 | 0.033 | 0.88 |
|  |  | Industry sponsorship | 0.034 | 0.035 | 0.32 |
|  |  | Overlapping population | -0.019 | 0.047 | 0.69 |
|  |  | Immunosuppressed population | 0.016 | 0.032 | 0.48 |
| A(H3N2) | ΔSCR | Sample size <100 | 0.056 | 0.079 | 0.48 |
|  |  | Low risk of bias | 0.049 | 0.068 | 0.47 |
|  |  | Industry sponsorship | -0.081 | 0.063 | 0.19 |
|  |  | Overlapping population | 0.036 | 0.086 | 0.67 |
|  |  | Immunosuppressed population | 0.121 | 0.056 | 0.030 |
|  | ΔSPR | Sample size <100 | 0.024 | 0.059 | 0.41 |
|  |  | Low risk of bias | 0.013 | 0.050 | 0.79 |
|  |  | Industry sponsorship | 0.007 | 0.051 | 0.90 |
|  |  | Overlapping population | -0.026 | 0.063 | 0.68 |
|  |  | Immunosuppressed population | 0.049 | 0.046 | 0.29 |
| B | ΔSCR | Sample size <100 | 0.034 | 0.065 | 0.60 |
|  |  | Low risk of bias | 0.001 | 0.050 | 0.99 |
|  |  | Industry sponsorship | -0.047 | 0.046 | 0.30 |
|  |  | Overlapping population | 0.000 | 0.059 | 0.99 |
|  |  | Immunosuppressed population | 0.075 | 0.042 | 0.071 |
|  | ΔSPR | Sample size <100 | 0.007 | 0.056 | 0.91 |
|  |  | Low risk of bias | 0.054 | 0.038 | 0.15 |
|  |  | Industry sponsorship | -0.029 | 0.037 | 0.44 |
|  |  | Overlapping population | 0.007 | 0.056 | 0.91 |
|  |  | Immunosuppressed population | 0.003 | 0.039 | 0.95 |

SCR, seroconversion rate; SPR, seroprotection rate; ΔSCR: difference in seroconversion rates between subjects immunized with adjuvanted vs non-adjuvanted influenza vaccines; ΔSPR: difference in seroprotection rates between subjects immunized with adjuvanted vs non-adjuvanted influenza vaccines.
