# Supplementary material for: Immunogenicity and safety of the MF59-adjuvanted seasonal influenza vaccine in non-elderly adults: A systematic review and meta-analysis
Source: PLoS One. 2024 Dec 30;19(12):e0310677. doi: 10.1371/journal.pone.0310677 (PMC11684710; doi:10.1371/journal.pone.0310677)
Supplement: S10 Table — (DOCX) [file pone.0310677.s056.docx]

**S10 Table. Extracted data on the comparison of seroconversion and seroprotection rates and geometric mean titer ratios against heterologous strains 3–4 weeks after one dose of the MF59-adjuvanted or non-adjuvanted seasonal influenza vaccines in non-elderly adults, by strain, presence of immunosuppressive conditions and serological parameter.**

| **Vaccine strain (subtype/lineage)** | **Heterologous strain (subtype/lineage)** | **Seroconversion rate, % (n/N)** | | | **Seroprotection rate, % (n/N) ^a^** | | | **GMTR aTIV/aQIV vs. TIV/QIV (p)** | **Ref** |
| --- | --- | --- | --- | --- | --- | --- | --- | --- | --- |
|  |  | **aTIV** | **TIV** | **p** | **aTIV/aQIV** | **TIV/QIV** | **p** |  |  |
| A/New Caledonia/20/1999 (H1N1) | A/Italy/05/2009 (H1N1pdm09) | 19.2 (5/26) | NA | NA | 30.7 (8/26) | NA | NA | NA | [64] |
| A/Solomon Islands/3/2006 (H1N1) | A/Italy/05/2009 (H1N1pdm09) | 12.5 (2/16) | NA | NA | 12.5 (2/16) | NA | NA | NA | [52] |
| A/New Caledonia/20/1999 (H1N1) | A/Solomon Islands/3/2006 (H1N1) | 48 (78/163) | 35 (55/156) | 0.018 | 67 (109/163) | 56 (87/156) | ns | 1.62 (0.013) | [65] |
| A/California/7/2009 (H1N1pdm09) | A/New Caledonia/20/1999 (H1N1) | 9.7 (3/31) | 24.1 (7/29) | 0.18 | 90.3 (28/31) | 86.2 (25/29) | 0.62 | 1.33 (0.48) | [55] |
| A/New York/55/2004 (H3N2) | A/Wisconsin/67/2005 (H3N2) | 67.5 (81/120) | 50.8 (60/118) | 0.008 | 79.2 (95/120) | 61.0 (72/118) | 0.002 | 1.58 (0.001) | [51] |
| A/Wisconsin/67/2005 (H3N2) | A/California/7/2004 (H3N2) | 74 (121/163) | 66 (103/156) | ns | 96 (156/163) | 91 (142/156) | ns | 1.90 (<0.001) | [65] |
| A/Victoria/361/  2011 (H3N2) | A/Texas/50/2012  (H3N2) | 41.9 (13/31) | 41.4 (12/29) | 0.97 | 83.9 (26/31) | 72.4 (21/29) | 0.28 | 1.24 (0.71) | [55] |
| B/Jiangsu/10/2003 (B/Yamagata) | B/Malaysia/2506/2004 (B/Victoria) | 10.0 (12/120) | 9.3 (11/118) | ns | 39.2 (47/120) | 42.4 (50/118) | ns | 1.03 (ns) | [51] |
| B/Malaysia/2506/2004 (B/Victoria) | B/Shanghai/361/2002 (B/Yamagata) | 40 (65/163) | 35 (55/156) | ns | 62 (101/163) | 53 (83/156) | ns | 1.45 (0.022) | [65] |
| B/Wisconsin/1/2010 (B/Yamagata) | B/Brisbane/60/2008 (B/Victoria) | 16.1 (5/31) | 10.3 (3/29) | 0.71 | 41.9 (13/31) | 34.5 (10/29) | 0.55 | 1.05 (0.98) | [55] |
| B/Hong Kong/330/2001 (B/Victoria) | B/Malaysia/2506/2004 (B/Victoria) | NA | NA | NA | 92.3 (24/26) | NA | NA | NA | [47] |
| B/Hong Kong/330/2001 (B/Victoria) | B/Sichuan/379/1999 (B/Yamagata) | NA | NA | NA | 88.5 (23/26) | NA | NA | NA | [47] |
| B/Hong Kong/330/2001 (B/Victoria) | B/Shanghai/361/2002 (B/Yamagata) | NA | NA | NA | 38.5 (10/26) | NA | NA | NA | [47] |

^a^Hemagglutination inhibition titer ≥ 1:40.

aQIV, quadrivalent MF59-adjuvanted seasonal influenza vaccine; aTIV, trivalent MF59-adjuvanted seasonal influenza vaccine; QIV, quadrivalent non-adjuvanted seasonal influenza vaccine; TIV, trivalent non-adjuvanted seasonal influenza vaccine; GMTR, geometric mean titer ratio; ns, non-significant at p<0.05; NA, not available.
