# Supplementary material for: Immunogenicity and safety of the MF59-adjuvanted seasonal influenza vaccine in non-elderly adults: A systematic review and meta-analysis
Source: PLoS One. 2024 Dec 30;19(12):e0310677. doi: 10.1371/journal.pone.0310677 (PMC11684710; doi:10.1371/journal.pone.0310677)
Supplement: S11 Table — (DOCX) [file pone.0310677.s057.docx]

**S11 Table. Meta-analysis of relative seroconversion and seroprotection rates towards heterologous strains 3–4 weeks after one dose of the MF59-adjuvanted or non-adjuvanted seasonal influenza vaccines in non-elderly adults, by heterologous strain.**

| **Parameter** | **Heterologous strain** | **k** | **I^2^, %** | **FE model, % (95% CI)** | **RE model, % (95% CI)** |
| --- | --- | --- | --- | --- | --- |
| ΔSCR | A(H1N1) | 2 | 83.4 | 8.3 (-1.2, 17.8) | 0.2 (-26.2, 26.2) |
|  | A(H3N2) | 3 | 0.0 | 10.7 (3.2, 18.2) | 10.6 (3.2, 18.0) |
|  | B | 3 | 0.0 | 3.2 (-3.2, 9.6) | 2.4 (-3.3, 8.2) |
| ΔSPR | A(H1N1) | 2 | 0.0 | 10.0 (0.7, 19.3) | 9.0 (0.1, 17.9) |
|  | A(H3N2) | 3 | 55.6 | 10.5 (4.9, 16.1) | 10.2 (0.5, 19.9) |
|  | B | 3 | 4.8 | 4.0 (-0.4, 11.8) | 3.9 (-5.4, 13.2) |

FE, fixed effects; RE, random effects; ΔSCR: difference in seroconversion rates between subjects immunized with adjuvanted vs non-adjuvanted influenza vaccines; ΔSPR: difference in seroprotection rates between subjects immunized with adjuvanted vs non-adjuvanted influenza vaccines.
