# Supplementary material for: Immunogenicity and safety of the MF59-adjuvanted seasonal influenza vaccine in non-elderly adults: A systematic review and meta-analysis
Source: PLoS One. 2024 Dec 30;19(12):e0310677. doi: 10.1371/journal.pone.0310677 (PMC11684710; doi:10.1371/journal.pone.0310677)
Supplement: S12 Table — (DOCX) [file pone.0310677.s058.docx]

**S12 Table. Extracted data on the comparison of seroprotection rates and geometric mean titer ratios towards vaccine-like strains 3–9 months after one dose of the MF59-adjuvanted or non-adjuvanted seasonal influenza vaccines in non-elderly adults, by strain, immunosuppression status and time post-vaccination.**

| **Virus** | **Time, months** | **Immunosuppression** | **Seroprotection rate, % (n/N)** | | | **GMTR aTIV/aQIV vs. TIV/QIV (95% CI or p)** | **Ref** |
| --- | --- | --- | --- | --- | --- | --- | --- |
|  |  |  | **aTIV/aQIV** | **TIV/QIV** | **p** |  |  |
| A(H1N1) | 3 | No | 93 (69/74)^a^ | 91 (60/66)^a^ | ns | 1.81 (NR) | [48] |
|  | 3 | Yes | 90 (28/31)^a^ | 81 (39/48)^a^ | ns | 1.72 (NR) | [48] |
|  | 6 | No | 73 (NR)^b^ | 57 (NR)^b^ | 0.025 | NR | [42] |
|  | 6 | Yes | 50 (9/18)^a^ | 74 (14/19)^a^ | ns | NR | [46] |
|  | 6 | No | 100 (14/14) | 71.4 (10/14) | ns | 2.56 (<0.05) | [56] |
|  | 6 | Yes | 65.5 (38/58) | 57.7 (30/52) | 0.40 | 1.55 (0.08) | [59] |
|  | 6 | Yes | 80 (153/192) | 73 (140/191) | ns | 1.43 (0.005) | [62] |
|  | 6 | No | 98.2 (1009/1027) | 96.3 (978/1016) | <0.05 | 1.15 (1.06; 1.25) | [63] |
|  | 9 | No | NR | NR | NR | 1.12 (1.02; 1.23) | [63] |
| A(H3N2) | 3 | No | 95 (70/74)^a^ | 98 (65/66)^a^ | ns | 0.78 (NR) | [48] |
|  | 3 | Yes | 90 (28/31)^a^ | 83 (40/48)^a^ | ns | 1.13 (NR) | [48] |
|  | 6 | No | NR^b^ | NR^b^ | ns | NR | [42] |
|  | 6 | Yes | 55 (10/18)^a^ | 47 (9/19)^a^ | ns | NR | [46] |
|  | 6 | No | 92.9 (13/14) | 71.4 (10/14) | ns | 2.56 (ns) | [56] |
|  | 6 | Yes | 89.7 (52/58) | 84.6 (44/52) | 0.43 | 1.24 (0.31) | [59] |
|  | 6 | Yes | 60 (116/192) | 52 (99/191) | ns | 1.16 (0.26) | [62] |
|  | 6 | No | 92.0 (945/1027) | 89.9 (913/1016) | ns | 1.05 (0.97; 1.14) | [63] |
|  | 9 | No | NR | NR | NR | 1.06 (0.98; 1.14) | [63] |
| B/Victoria | 6 | Yes | 50 (9/18)^a^ | 58 (11/19)^a^ | ns | NR | [46] |
|  | 6 | Yes | 43 (83/192) | 32 (61/191) | <0.05 | 1.21 (0.10) | [62] |
|  | 6 | No | 83.4 (857/1027) | 84.3 (856/1016) | ns | 0.97 (0.91; 1.04) | [63] |
|  | 9 | No | NR | NR | NR | 0.98 (0.92; 1.03) | [63] |
| B/Yamagata | 3 | No | 95 (70/74)^a^ | 94 (62/66)^a^ | ns | 1.26 (NR)^c^ | [48] |
|  | 3 | Yes | 90 (28/31)^a^ | 90 (43/48)^a^ | ns | 0.79 (NR)^c^ | [48] |
|  | 6 | No | NR^b^ | NR^b^ | ns | NR | [42] |
|  | 6 | No | 64.3 (9/14) | 42.9 (6/14) | ns | 2.62 (<0.05) | [56] |
|  | 6 | Yes | 77.6 (45/58) | 76.9 (40/52) | 0.93 | 1.18 (0.32)^c^ | [59] |
|  | 6 | No | 83.9 (862/1027) | 84.0 (853/1016) | ns | 0.99 (0.93; 1.05)^d^ | [63] |
|  | 9 | No | NR | NR | NR | 0.98 (0.93; 1.03)^d^ | [63] |

^a^ HAI titer ≥1:40; ^b^ HAI titer ≥1:160.

aQIV, quadrivalent MF59-adjuvanted seasonal influenza vaccine; aTIV, trivalent MF59-adjuvanted seasonal influenza vaccine; QIV, quadrivalent non-adjuvanted seasonal influenza vaccine; TIV, trivalent non-adjuvanted seasonal influenza vaccine; GMTR, geometric mean titer ratio; ns, non-significant at p <0.05; NR, not reported.
