# Supplementary material for: Immunogenicity and safety of the MF59-adjuvanted seasonal influenza vaccine in non-elderly adults: A systematic review and meta-analysis
Source: PLoS One. 2024 Dec 30;19(12):e0310677. doi: 10.1371/journal.pone.0310677 (PMC11684710; doi:10.1371/journal.pone.0310677)
Supplement: S13 Table — (DOCX) [file pone.0310677.s059.docx]

**S13 Table. Meta-analysis of absolute and relative seroprotection rates towards vaccine-like strains six months after one dose of the MF59-adjuvanted or non-adjuvanted seasonal influenza vaccines in non-elderly adults, by strain and time post-vaccination.**

| **Parameter** | **Vaccine-like strain** | **k** | **I^2^, %** | **FE model, % (95% CI)** | **RE model, % (95% CI)** |
| --- | --- | --- | --- | --- | --- |
| SPR | A(H1N1) | 6 | 97.5 | 95.1 (93.8, 96.2) | 82.0 (63.4, 95.3) |
|  | A(H3N2) | 5 | 96.4 | 89.1 (87.2, 90.8) | 80.5 (63.0, 93.6) |
|  | B | 5 | 96.9 | 78.3 (75.9, 80.6) | 65.4 (47.4, 81.5) |
| ΔSPR | A(H1N1) | 6 | 65.7 | 4.1 (2.1, 6.2) | 6.9 (-0.2, 14.0) |
|  | A(H3N2) | 5 | 0.0 | 3.5 (1.0, 6.1) | 4.2 (-0.3, 8.6) |
|  | B | 5 | 43.0 | 1.2 (-1.8, 4.2) | 3.3 (-4.2, 10.8) |

FE, fixed effects; RE, random effects; SPR, seroprotection rate; ΔSPR: difference in seroprotection rates between subjects immunized with adjuvanted vs non-adjuvanted influenza vaccines.
