# Supplementary material for: Immunogenicity and safety of the MF59-adjuvanted seasonal influenza vaccine in non-elderly adults: A systematic review and meta-analysis
Source: PLoS One. 2024 Dec 30;19(12):e0310677. doi: 10.1371/journal.pone.0310677 (PMC11684710; doi:10.1371/journal.pone.0310677)
Supplement: S14 Table — (DOCX) [file pone.0310677.s060.docx]

**S14 Table. Extracted data on the comparison of local/injection site and systemic solicited adverse reactions during the first week after one dose of the MF59-adjuvanted or non-adjuvanted seasonal influenza vaccines in non-elderly adults, by adverse reaction.**

| **Adverse reaction** | | **Study [Ref]** | | | | | | | |
| --- | --- | --- | --- | --- | --- | --- | --- | --- | --- |
|  |  | **Frey 2003 [42]^a^** | | **Pollok 2004 [45]^a^** | | **Gabutti 2005 [46]^b^** | | **Durando 2008 [48]^b^** | |
|  |  | **aTIV (N=150)** | **TIV (N=151)** | **aTIV (N=60)** | **TIV (N=56)** | **aTIV (N=18)** | **TIV (N=19)** | **aTIV (N=127)** | **TIV (N=129)** |
| Local | Any | NA | NA | NA | NA | 27.8 (5) | 10.5 (2) | NA | NA |
|  | Pain | 90.0 (135) | 63.6 (96) | 52 (31) | 20 (11) | NA | NA | 47.2 (60) | 21.7 (28) |
|  | Erythema | 19.3 (29) | 21.9 (33) | NA | NA | NA | NA | 7.1 (9) | 3.9 (5) |
|  | Induration | 22.0 (33) | 17.2 (26) | NA | NA | NA | NA | 20.5 (26) | 7.8 (10) |
|  | Ecchymosis | NA | NA | NA | NA | NA | NA | 2.4 (3) | 0.8 (1) |
| Systemic | Any | NA | NA | NA | NA | 5.6 (1) | 10.5 (2) | NA | NA |
|  | Fever | 0.7 (1) | 0 (0) | NA | NA | 5.6 (1) | 10.5 (2) | 26.8 (34) | 4.7 (6) |
|  | Chills | 4.7 (7) | 0.7 (1) | NA | NA | NA | NA | NA | NA |
|  | Myalgia | 14.7 (22) | 6.0 (9) | 14 (8) | 4 (2) | NA | NA | 14.2 (18) | 5.4 (7) |
|  | Arthralgia | 2.7 (4) | 0 (0) | NA | NA | NA | NA | 12.6 (16) | 7.0 (9) |
|  | Headache | 22.7 (34) | 20.5 (31) | NA | NA | NA | NA | 27.6 (35) | 14.7 (19) |
|  | Malaise | 9.3 (14) | 7.9 (12) | NA | NA | NA | NA | 32.3 (41) | 17.1 (22) |
|  | Nausea | 2.0 (3) | 2.0 (3) | NA | NA | NA | NA | NA | NA |
|  | Rash | 0 (0) | 0.7 (1) | NA | NA | NA | NA | NA | NA |
| **Adverse reaction** | | **Study [Ref]** | | | | | | | |
|  |  | **Baldo 2007 [50]^a^** | | **Baldo 2012 [65]^a^** | | **Kumar 2016 [54]^a^** | | **Natori 2017 [60]^a^** | |
|  |  | **aTIV (N=128)** | **TIV (N=128)** | **aTIV (N=180)** | **TIV (N=179)** | **aTIV (N=31)** | **TIV (N=31)** | **aTIV (N=35)** | **TIV (N=38)** |
| Local | Any | 46.9 (60) | 24.2 (31) | 49 (89) | 28 (50) | NA | NA | NA | NA |
|  | Pain | 28.9 (37) | 8.6 (11) | 45 (81) | 20 (36) | 77.4 (24) | 51.6 (16) | NA | NA |
|  | Erythema | 12.5 (16) | 4.7 (6) | 4 (7) | 8 (14) | 9.7 (3) | 3.2 (1) | NA | NA |
|  | Induration | 10.2 (13) | 7.8 (10) | 13 (24) | 8 (15) | 6.5 (2) | 6.5 (2) | NA | NA |
|  | Ecchymosis | 3.1 (4) | 2.3 (3) | 7 (13) | 8 (14) | NA | NA | NA | NA |
| Systemic | Any | 25.8 (33) | 18.8 (24) | 49 (89) | 28 (50) | NA | NA | NA | NA |
|  | Fever | 3.9 (5) | 3.9 (5) | 1 (1) | 0 (0) | 0 (0) | 0 (0) | 5.7 (2) | 2.6 (1) |
|  | Chills | 7.8 (10) | 6.3 (8) | 8 (15) | 9 (17) | NA | NA | NA | NA |
|  | Myalgia | 7.8 (10) | 7.0 (9) | 31 (55) | 9 (17) | NA | NA | NA | NA |
|  | Arthralgia | 0.8 (1) | 0 (0) | 18 (33) | 13 (23) | NA | NA | NA | NA |
|  | Headache | 7.8 (10) | 7.8 (10) | 21 (37) | 17 (30) | NA | NA | NA | NA |
|  | Malaise | 18.8 (24) | 14.1 (18) | 22 (40) | 13 (23) | NA | NA | NA | NA |
|  | Nausea | 4.7 (6) | 3.1 (4) | NA | NA | NA | NA | NA | NA |
|  | Rash | 0 (0) | 0 (0) | NA | NA | NA | NA | NA | NA |
| **Adverse reaction** | |  | | | | | | | |
|  |  | **Kazmin 2023 [61]^c^** | | **Mombeli 2024 [62]^a^** | | | **Poder 2023 [63]^a^** | | |
|  |  | **aTIV (N=31)** | | **aTIV (N=209)** | **QIV (N=204)** | **hdTIV (N=203)** | **aQIV (N=1,020)** | **QIV (N=1,008)** | |
| Local | Any | NA | | NA | NA | NA | 49.8 (508) | 30.4 (306) | |
|  | Pain | 16 (5) | | 51 (106) | 22 (45) | 41 (84) | 47.1 (480) | 28.1 (283) | |
|  | Erythema | NA | | 12 (26) | 8 (16) | 11 (23) | 7.8 (80) | 3.1 (31) | |
|  | Induration | NA | | NA | NA | NA | 7.9 (81) | 3.5 (35) | |
|  | Ecchymosis | NA | | NA | NA | NA | 0.6 (6) | 0.6 (6) | |
| Systemic | Any | NA | | NA | NA | NA | 45.3 (462) | 40.0 (403) | |
|  | Fever | 0 (0) | | 6 (12) | 2 (5) | 7 (15) | 2.5 (26) | 1.7 (17) | |
|  | Chills | NA | | NA | NA | NA | 6.6 (67) | 5.5 (55) | |
|  | Myalgia | NA | | 16 (33) | 14 (29) | 19 (38) | 13.0 (133) | 7.2 (73) | |
|  | Arthralgia | NA | | 12 (26) | 9 (19) | 10 (21) | 13.7 (140) | 9.4 (95) | |
|  | Headache | NA | | 18 (37) | 15 (31) | 25 (50) | 22.2 (226) | 20.4 (206) | |
|  | Malaise | NA | | NA | NA | NA | NA | NA | |
|  | Nausea | NA | | 6 (12) | 4 (8) | 11 (22) | 7.3 (74) | 4.4 (44) | |
|  | Rash | NA | | NA | NA | NA | NA | NA | |

^a^Solicited adverse reactions were collected during the first seven days post-vaccination; ^b^Solicited adverse reactions were collected during the first four days post-vaccination; ^c^Solicited adverse reactions were collected during the first three days post-vaccination;

aQIV, quadrivalent MF59-adjuvanted seasonal influenza vaccine; aTIV, trivalent MF59-adjuvanted seasonal influenza vaccine; hdTIV, high-dose seasonal influenza vaccine; QIV, quadrivalent non-adjuvanted seasonal influenza vaccine; TIV, trivalent non-adjuvanted seasonal influenza vaccine; NA, not available.
